# Early Life Wheeze and Risk Factors for Asthma—A Revisit at Age 7 in the GEWAC-Cohort

**DOI:** 10.3390/children8060488

**Published:** 2021-06-08

**Authors:** Idun Holmdahl, Anastasia Filiou, Katarina Stenberg Hammar, Anna Asarnoj, Magnus P. Borres, Marianne van Hage, Gunilla Hedlin, Cilla Söderhäll, Jon R. Konradsen

**Affiliations:** 1 Astrid Lindgren’s Children’s Hospital, Karolinska University Hospital, 171 64 Stockholm, Sweden; anastasia.filiou@ki.se (A.F.); Katarina.Stenberg@bsmartina.se (K.S.H.); anna.asarnoj@ki.se (A.A.); gunilla.hedlin@ki.se (G.H.); cilla.soderhall@ki.se (C.S.); Jon.konradsen@ki.se (J.R.K.); 2 Department of Women’s and Children’s Health, Karolinska Institutet, 171 77 Stockholm, Sweden; 3 Department of Women’s and Children’s Health, Uppsala University, 752 36 Uppsala, Sweden; Magnus.borres@thermofisher.com; 4 Department of Medicine Solna, Division of Immunology and Allergy, Karolinska Institutet and Karolinska University Hospital, 171 76 Stockholm, Sweden; marianne.van.hage@ki.se

**Keywords:** preschool wheeze, rhinovirus, allergic sensitization, asthma, wheeze

## Abstract

One third of all toddlers are in need of medical care because of acute wheeze and many of these children have persistent asthma at school age. Our aims were to assess risk factors for and the prevalence of asthma at age 7 in a cohort of children suffering from an acute wheezing episode as toddlers. A total of 113 children, included during an acute wheezing episode (cases), and 54 healthy controls were followed prospectively from early pre-school age to 7 years. The protocol included questionnaires, ACT, FeNO, nasopharyngeal virus samples, blood sampling for cell count, vitamin D levels, and IgE to food and airborne allergens. The prevalence of asthma at age 7 was 70.8% among cases and 1.9% among controls (*p* < 0.001). Acute wheeze caused by rhinovirus (RV) infection at inclusion was more common among cases with asthma at age 7 compared to cases without asthma (*p* = 0.011) and this association remained significant following adjustment for infection with other viruses (OR 3.8, 95% CI 1.4–10.5). Cases with asthma at age 7 had been admitted to hospital more often (*p* = 0.024) and spent more days admitted (*p* = 0.01) during the year following inclusion compared to cases without asthma. RV infection stands out as the main associated factor for wheeze evolving to persistent asthma. Cases who developed asthma also had an increased need of hospital time and care for wheeze during the year after inclusion.

## 1. Introduction

One third of all toddlers suffer from pre-school wheeze and approximately 40% of these children will continue to have recurrent wheeze and asthma at school age [1,2,3]. Multiple risk factors for recurrent wheeze and asthma have been identified including allergic sensitization, eczema and atopy, parental smoking and other environmental exposures, low levels of vitamin D, and a family history of asthma [4,5,6].

One of the strongest risk factors for recurrent wheeze and asthma appears to be viral respiratory infections. Rhinovirus (RV)-induced infection triggers the innate immune response in susceptible individuals, which causes disrupted tight junctions in the airway epithelium and high cytokine levels that promote bronchospasm, edema, mucus secretion, and airway hyperresponsiveness [7,8]. Early RV-induced wheezing has been shown to be an important risk factor for recurrent wheeze and doctor-diagnosed asthma at the ages from 6 to 13 years [9]. Respiratory syncytial virus (RSV)-induced lower respiratory tract illnesses (LRIs) are also associated with recurrent wheeze but the risk of asthma development decreases markedly with age and is not significant by the age of 13 [10].

Previous results from the GEWAC cohort (Gene Expression in Wheezing and Asthmatic Children) have demonstrated that risk alleles in the RV-C receptor CDHR3 [11], RV-strains, immunological response [12], environmental exposure, and comorbidities are all contributors to the clinical manifestation of the acute wheezing episode [13].

In the current GEWAC study, we followed 113 children suffering from an acute wheezing episode (cases) and 54 healthy controls prospectively from early pre-school age to 7 years. The aim was to assess the prevalence of and risk factors for asthma at age 7 through a detailed assessment of clinical characteristics, etiology of viral wheeze, inflammatory markers in blood and exhaled air, allergic sensitization, and measurement of pulmonary function.

## 2. Materials and Methods

### 2.1. Study Design and Cohort

**Figure 1:**
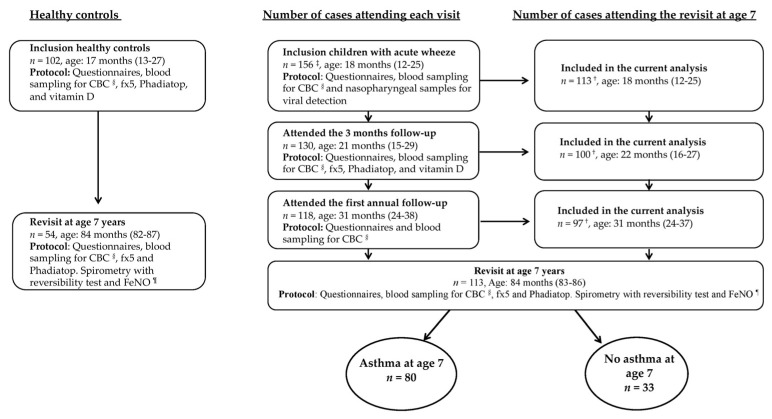
Flow-chart of number of controls and cases that attended the different follow-ups and sample collections at each visit. Age in months presented as medians with inter-quartile ranges (IQR). ^†^ Number of cases that attended the revisit at age 7 years. ^‡^ Two cases were excluded after inclusion because of prematurity and pseudo croup. ^§^ CBC = complete blood count. ^¶^ Nitric oxide in exhaled air.

The GEWAC (Gene Expression in Wheezing and Asthmatic Children) study is a longitudinal cohort study of 156 children (age 6–48 months) recruited during an acute wheezing episode from the pediatric emergency ward at Astrid Lindgren’s Children’s Hospital, Stockholm, Sweden, between 2008 and 2012 (cases). During the same time period, 102 age-matched healthy controls were recruited from the surgical day-care ward [12,13]. Exclusion and inclusion criteria are found in Supplementary Materials Table S1. The cases were invited to follow-ups after 2–3 months, one year and at age 7 years. The study population in the current analysis consists of the 113 cases and 54 healthy controls who attended the revisit at age 7 years, Figure 1. Seven of the cases and 14 of the healthy controls did not physically attend the revisit at age 7 years but their legal guardian was interviewed by telephone.

### 2.2. Sample Collection

A standardized questionnaire regarding demography, parental history of asthma and allergies, breastfeeding, exposure to tobacco smoke and pets, pre-school attendance, previous infections and wheeze, occurrence of atopic and asthmatic symptoms and use of asthma medication was filled out at the first revisit. Symptoms of asthma and atopy, triggering factors, use of asthma medication and utilization of health care services were assesses at the following revisits along with the Asthma Control Test (ACT) at age 7. The legal guardians were advised not to give their children asthma medication (salbutamol, corticosteroids, or leukotriene antagonists) up to 24 h before the revisit at 7 years of age.

Nasopharyngeal swabs for the viral detection of enterovirus, RSV, adenovirus, bocavirus, coronavirus (229E, HKU1, NL63, OC43), influenza (A, H1N1, B), metapneumovirus, and parainfluenzavirus (1, 2, 3) were taken at the emergency visit and analyzed as previously described [13]. Children enrolled from September 2010 (*n* = 88) were also tested for bacteria at the emergency visit.

Blood samples were collected at each visit and analyzed for complete blood count. Cut-off levels for eosinophils and neutrophils were >0.3 × 10^9^/L and >3.25 × 10^9^/L, respectively, and calculated based on medians from the revisit at 7 years of age. Levels of vitamin D 25 (OH) were measured at the 3-month follow-up. Allergen-specific IgE antibodies against a mix of common food allergens, fx5 (milk, egg white, wheat, codfish, peanut, and soya bean), and a mix of common airborne allergens, Phadiatop (house dust mite, cat, horse, dog, timothy, birch, and mugwort, *Cladosporium herbarum*), were analyzed using the ImmunoCAP System (Thermo Fisher Scientific, Uppsala, Sweden) at the 3-month visit and at the revisit at age 7 years. Allergic sensitization was defined as allergen-specific IgE ≥ 0.35 kU_A_/L. Airborne allergy and food allergy were defined as having both reported symptoms and confirmed allergic sensitization. The blood samples were analyzed at the laboratories of Clinical Chemistry and of Clinical Immunology, Karolinska University Hospital, Stockholm Sweden.

To assess airway inflammation at the revisit at 7 years of age FeNO (fractioned exhaled nitric oxide) measurements were performed, using the non-invasive apparatus NIOX VERO (Circassia AB, Uppsala, Sweden).

Spirometry and airway reversibility were tested using the Medikro Pro spirometer (Medikro Oy, Kuopio, Finland). Reversibility was evaluated 15 min after inhalation with 400 micrograms salbutamol using a spacer. The patient’s technique and quality of the spirometry were evaluated according to international guidelines [14], and if the test criteria were not fulfilled, the test result was not included in the analyses.

### 2.3. Asthma Definition

The definition of asthma at 7 years of age was based on GINA guidelines [15] and included one compulsory criteria (a diagnosis of asthma by the study doctor, a pediatric allergist (KSH)) and three additional criteria: lower respiratory symptoms, medication for treatment of wheeze in the preceding 12 months, or airway reversibility >12% after the use of a bronchodilator with salbutamol, Supplementary Materials Table S2. Lower respiratory symptoms were defined as cough, shortness of breath, or nocturnal awakening caused by wheeze for 5 days or longer in the preceding 12 months. All children that fulfilled the compulsory and at least one of the additional criteria (symptoms, medication, or reversibility) were classified as having asthma.

Children with a doctor’s diagnosis of asthma were diagnosed by different doctors, before or after inclusion in the study according to national guidelines.

### 2.4. Statistical Analyses

SPSS version 25(IBM) was used. Two-tailed probability of <0.05 was considered statistically significant. The Chi-square test was used to examine significant proportional differences between two groups and Fisher’s exact test was used on small sample sizes. An unpaired *t*-test was used for continuous variables with normal distribution and a nonparametric test, Mann–Whitney, was used for skewed continuous variables. Univariate and multivariate logistic regression with adjustment for different viruses was performed. We calculated the risk of asthma at age 7 using admissions to hospital and days spent admitted to hospital because of wheeze as exposures in an unadjusted univariate logistic regression model.

## 3. Results

The study population is composed of 113 cases and 54 controls that attended the revisit at 7 years of age in the GEWAC cohort, Figure 1. No differences were seen between cases who attended the revisit at age 7 years (*n* = 113) and those that did not attend the revisit at age 7 years (*n* = 41), Supplementary Materials Table S3.

### 3.1. Cases Compared to Controls

Baseline characteristics of cases and controls that attended the revisit at 7 years of age are shown in Table 1. At age 7, the majority of the cases had asthma (70.8%) in comparison to 1.9% of the control group (*p* < 0.001) and cases had a higher prevalence of airborne allergy compared to controls (16.7% vs. 0%, *p* = 0.026). However, there was no difference in the prevalence of allergic sensitization between the two groups neither at the 3-month follow-up visit (*p* = 0.17 and *p* = 0.70) nor at the revisit at 7 years of age (*p* = 0.072), Table 1.

### 3.2. Cases with Asthma Compared to Cases without Asthma at Age 7 Years

**Figure 2:**
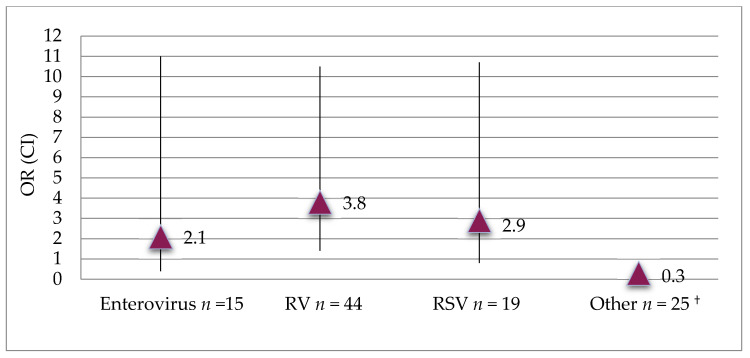
The association between infection with different viruses at inclusion and asthma at age 7 years. ^†^ Adenovirus, bocavirus, coronavirus (229E, HKU1, NL63, OC43), influenza (A, H1N1, B), metapneumovirus, and parainfluenzavirus (1, 2, 3).

The cases were divided into two groups based on whether or not they had asthma at 7 years of age, Table 2. Acute wheeze caused by RV infection at inclusion was more common among cases with asthma at age 7 compared to cases without asthma (48.1% vs. 21.9%, *p* = 0.011 and OR 3.3, 95% CI 1.3–8.5) and this association was significant after adjustment for infection with other viruses (OR 3.8, 95% CI 1.4–10.5), Figure 2. In contrast, wheezing caused by RSV at inclusion was not associated with asthma at age 7 (OR 2.9 95% CI 0.8–10.7). The combination of acute wheeze not caused by RV and no early aeroallergen sensitization was more common among children without asthma compared to those with asthma at age 7 (72.4% vs. 47.8%, *p* = 0.026 and OR 0.3, 95% CI 0.14–0.9). Recurrent wheeze at inclusion was not associated with asthma at age 7 (78.6% vs. 80.0%, *p* = 0.87 and OR 1.1, CI 0.3–3.2).

**Figure 3:**
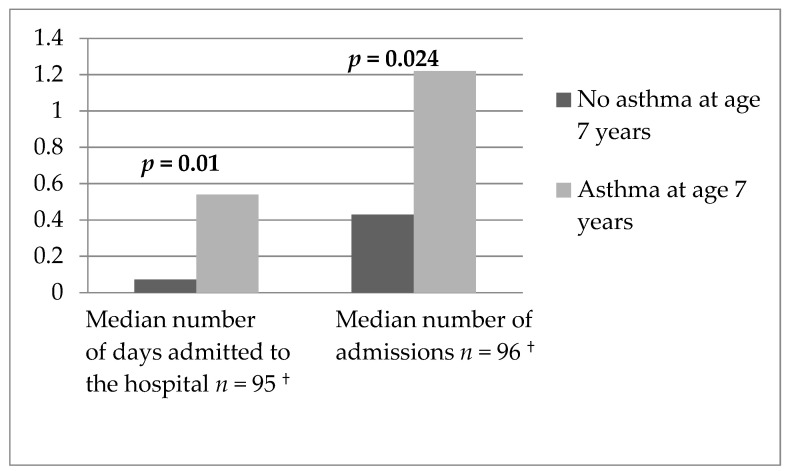
Number of days hospitalized for respiratory difficulties and number of admissions during the year following inclusion in the study. Data collected at the first annual follow-up. ^†^ Number of cases in the analysis.

Cases with asthma at age 7 years were admitted to hospital more often because of respiratory difficulties during the year after inclusion in the study (*p* = 0.024) and spent more days admitted to the hospital during the same time (*p* = 0.01) in comparison to cases without asthma, Figure 3. Both the number of admissions as well as the number of days spent admitted to hospital due to wheeze were associated with an increased risk of asthma at age 7 years, OR 1.7 (95% CI 1.1–3.0) and OR 4.5 (95% CI 1.1–18.0), respectively.

At age 7, cases with asthma had more frequently high levels of blood eosinophils (54.2% vs. 29.2%, *p* = 0.038), lower scores at ACT (median 24 vs. 26.5, *p* < 0.001), and lower FEV% (median 87.3 vs. 91.2, *p* = 0.043) compared to cases without asthma. Furthermore, cases with asthma were more often allergic to airborne allergens (22.7% vs. 0%, *p* = 0.009) and reported a higher prevalence of food allergy (13.9% vs. 0%, *p* = 0.032) whereas no difference was seen regarding sensitization to food allergens (fx5), Table 2.

### 3.3. Comparison between Cases without Asthma and Healthy Controls

In contrast to the comparison between all cases and controls (Table 1), cases without asthma at age 7 and controls were similar with respect to heredity for asthma and allergy and had similar levels of vitamin D at inclusion, Table 3. Further, cases without asthma at age 7 reported a lower prevalence of food allergy (*p* = 0.04) and had a lower prevalence of positive fx5 (*p* = 0.029) compared to healthy controls. No differences were observed between the two groups regarding FEV% and eosinophils, Table 3. The remaining comparisons between cases without asthma and controls were similar to the comparison between all cases and controls.

## 4. Discussion

In this longitudinal case-control study, 113 children were included during an acute wheezing episode in early life and followed prospectively to age 7 years. We compared children with early wheeze and asthma at age 7 to children with early wheeze and no asthma at age 7 and also with healthy controls at age 7 years. The major findings were that the prevalence of asthma at age 7 was as high as 70.8% and that there is an association between RV-induced wheezing in early life and asthma at 7 years of age. Furthermore, cases with asthma at age 7 were admitted more often and spent more days admitted to the hospital because of respiratory difficulties during the year following inclusion compared to cases without asthma. Finally, we found that cases with asthma at age 7 had a higher prevalence of allergy to airborne allergens at the same age compared to cases without asthma at age 7.

The prevalence of asthma at 7 years of age among cases (70.8%) was higher compared to some previously published follow-up studies of children with pre-school wheeze [2,3,5]. One explanation could be that as many as 81% of the children in GEWAC were hospitalized at inclusion, which suggests that the majority had a severe wheezing episode at inclusion, a recognized risk factor for persistent asthma [16,17]. The prevalence of asthma in GEWAC is in line with other cohorts of children with a history of severe wheeze in early life [18]. As only 21% of the included children had a first-time wheezing episode and 52% already had a diagnosis of asthma before the first follow-up, GEWAC can be considered a high-risk cohort. Further, recurrent wheeze and asthma diagnosis in early life are also recognized as risk factors for subsequent asthma [19]. Other studies recruited children before birth and diagnosis of wheeze was obtained from outpatient clinics [20], which suggests less severe symptoms at inclusion or a more mixed group of wheezing children.

The asthma definition in this study is partly according to The Global Initiative for Asthma 2020 (GINA) [15] and based on the history of variable respiratory symptoms and evidence of variable expiratory airflow limitation. In addition, we added the use of medication with ICS and/or leukotriene antagonists for 5 days or longer in the previous 12 months, as such treatment may alter the presence and the intensity of symptoms. We also included spirometry and reversibility testing as they constitute a more objective measure of asthma than reported symptoms. Nevertheless, we are aware of the limitations of lung function testing, such as the results depending on the quality of the performed maneuvers, particularly in children at 7 years of age. Furthermore, the absence of reversibility does not rule out asthma [15]. For these reasons spirometry results constitute an additional criterion in our definition. Since respiratory symptoms are not pathognomonic for asthma and may suggest the presence of other conditions (e.g., malformations of the airway anatomy, allergic rhinitis, gastro esophageal reflux, cystic fibrosis, primary ciliary dyskinesia) [21], we added doctor’s diagnosis as a mandatory criterion in our definition.

The strong association between RV-induced wheeze and subsequent asthma confirms previous findings [22,23,24] and there are several potential mechanisms behind this association. Infection with RV can alter the epithelial barrier by disrupting the tight junctions between the epithelial cells, rendering the mucosa more vulnerable to allergen exposure and development of allergic sensitization [25]. Further, RV infection induces a release of airway innate cytokines (IL-25 and IL-33) that initiate type 2 differentiations of dendritic cells, T cells, and innate lymphoid cells, and subsequently the production of cytokines (IL-4 and IL-5) that starts and maintains an allergic inflammation [8]. The combined effect of the virus infection and the inflammatory response leads to further epithelial damage, mucus production, and edema resulting in airway obstruction and wheeze [26].

RV infection in genetically predisposed individuals can also alter the expression of many genes associated with immune response and may increase susceptibility to asthma [27]. In a genome-wide association study, a risk variant in the gene cadherin related family member 3 (*CDHR3*) was found associated with asthma exacerbations in early childhood [28]. *CDHR3* has a high level of expression in bronchial epithelium and is a receptor for rhinovirus C [29]. We have previously shown, in children from our GEWAC cohort, reduced mRNA levels of *CDHR3* in pre-school children with RV-induced wheeze [11].

Early life viral wheezing in combination with aeroallergen sensitization has been linked to an increased risk of developing asthma at school age [9]. We found that aeroallergen sensitization at 7 years of age was more common among cases with asthma compared to those without asthma. This finding is in line with the results published by Rubner et al. who showed that both early aeroallergen sensitization and RV-induced wheezing is associated with an increased risk of asthma, and if both are present, the risk is additive [9]. In addition, we found that the combination with acute wheeze not caused by RV and no early aeroallergen sensitization was more common among children without asthma at 7 years. Thus, children without indications of allergy and wheeze not caused by RV run a low risk of developing asthma at school age.

The strengths of this study are the prospective design and longitudinal clinical follow-up with clinical examinations, questionnaires, FeNO measurements, spirometry, and blood sampling. The detailed clinical characterization allowed multiple comparisons, between cases and controls, cases with asthma vs. cases without asthma at age 7 years, and healthy controls vs. cases without asthma at age 7 years, to explore the differences between each study group.

One limitation of the study is the sample size, which did not allow sub-group analyses of children with more severe respiratory symptoms and children with both sensitization and RV infection. Due to the small sample size, we had limited statistical power to test for potential confounders. Further, information about symptoms and medication was reported at the follow-up visits in standardized questionnaires, which presumably implies recall bias. Selection bias is possible with respect to children who attended the revisit at 7 years. However, the dropout analysis shows that there were no major differences at inclusion between those who attended and those who did not attend the revisit at 7 years. One of the exclusion criteria for the control group was known sensitization for airborne allergens; this may have given a selected control group.

## 5. Conclusions

Cases with asthma at age 7 were more often hospitalized and needed more inpatient time during the year after inclusion in the study compared to cases without asthma at age 7 years, which indicates that children with more severe wheezing symptoms have a higher risk of recurrent wheeze and later asthma. We confirmed that RV-induced acute wheeze in toddlers is associated with the development of asthma from pre-school wheeze. These findings indicate that nasopharyngeal virus sampling in pre-school children with acute wheeze is of importance to identify those who have a higher risk of developing subsequent asthma at school age.

## Figures and Tables

**Table 1:** Cases vs. control group that attended the revisit at age 7 years.

Variable	Cases *n* = 113	Controls *n* = 54	Statistics
**Demography/clinical history ^a^**			
Male, *n* (%)	73 (64.6)	39 (72.2)	*p* = 0.33
Age in months at inclusion, median (IQR)	18 (12–24.5)	16 (12.7–28.0)	*p* = 0.95
Caucasian father and/or mother, *n* (%)	96 (88.9)	44 (84.6)	*p* = 0.44
Maternal smoking during pregnancy, *n* (%)	9 (8.3)	2 (3.7)	*p* = 0.34
Parental heredity asthma and allergy, *n* (%)	79 (73.8)	26 (49.1)	*p* = 0.002
Exclusive breastfeeding 4 months, *n* (%)	66 (63.5)	34 (63.0)	*p* = 0.95
Attend childcare or family home day care at inclusion, *n* (%)	83 (76.9)	26 (48.1)	*p* < 0.001
>6 RTIs ^†^/year prior to inclusion, *n* (%)	69 (65.7)	6 (11.1)	*p* < 0.001
Previous RSV ^‡^ infection prior to inclusion, *n* (%)	30 (27.8)	2 (3.7)	*p* < 0.001
**Baseline characteristics ^b^**			
First time wheeze, *n* (%)	21 (21.0)	-	
Hospitalized acute, *n* (%)	81 (81.0)	-	
RTI ^†^ at the emergency visit	105 (92.9)	-	
Doctor’s diagnosis of asthma at 3-month follow-up, *n* (%)	52 (52.0)	-	
Reported food allergy at inclusion, *n* (%)	11(10.3)	1 (1.9)	*p* = 0.062
Eczema at inclusion, *n* (%)	24 (22.2)	2 (3.7)	*p* = 0.002
Positive Phadiatop (3-month follow-up), n (%)	10 (10.2)	1 (2.4)	*p* = 0.17
Positive fx5 (3-month follow-up), *n* (%)	22 (22.4)	8 (19.5)	*p* = 0.70
Vitamin D nmol/L, median (IQR)	83.0 (69–101)	96.0 (76–107.5)	*p* = 0.015
**Clinical data from the revisit at age 7 years ^c^**			
Age in months at the revisit at age 7 years, median (IQR)	84 (83.0–85.5)	84 (82.0–87.3)	*p* = 0.68
Asthma at age 7, *n* (%)	80 (70.8)	1 (1.9)	*p* < 0.001
Self-reported food allergy *n* (%)	11 (9.8)	7 (13.0)	*p* = 0.54
Self-reported airborne allergy *n* (%)	20 (18.2)	0 (0)	*p* = 0.001
FeNO, median (IQR)	9 (7–14)	10 (6–15)	*p* = 0.89
FEV%, median (IQR)	88.5 (82.3)	91.7 (85.8–98.2)	*p* = 0.069
Positive Phadiatop, *n* (%)	26 (28.9)	3 (11.5)	*p* = 0.072
Positive fx5, *n* (%)	19 (21.1)	10 (38.5)	*p* = 0.072
Airborne allergy (symptoms + sensitization)	15 (16.7)	0 (0)	*p* = 0.026
Food allergy (symptoms + sensitization)	7 (7.9)	2 (7.7)	*p* = 0.18
Eosinophils > 0.3 at the 7 years follow-up, *n* (%)	39 (47.0)	6 (24.0)	*p* = 0.041

^†^ Respiratory tract infection; ^‡^ respiratory syncytial virus; ^a^ controls *n* = 52–54, cases *n* = 104–113; ^b^ controls *n* = 41–54, cases *n* = 89–107; ^c^ controls *n* = 26–54, cases *n* = 90–113.

**Table 2:** Cases were divided into two groups based on whether they had asthma at the revisit at age 7 years or not.

Variable	Cases with Asthma at Age 7 *n* = 80	Cases without Asthma at Age 7 *n* = 33	Statistics
**Demography/clinical history ^a^**			
Male, *n* (%)	50 (62.5)	23 (69.7)	*p* = 0.47
Age in months at inclusion, median (IQR)	19 (12–25.7)	16 (13–23.0)	*p* = 0.68
Caucasian father and/or mother, *n* (%)	67 (88.2)	29 (90.6)	*p* = 0.71
Smoking during pregnancy mother, *n* (%)	8 (10.5)	1 (3.1)	*p* = 0.28
Parental heredity asthma and allergy, *n* (%)	57 (76.0)	22 (68.8)	*p* = 0.44
Exclusive breastfeeding 4 months, *n* (%)	44 (61.1)	22 (68.8)	*p* = 0.46
Attend childcare or family home day care at inclusion, *n* (%)	58 (76.3)	25 (78.1)	*p* = 0.84
>6 RTIs ^†^/year prior to inclusion, *n* (%)	47 (63.5)	22 (71.0)	*p* = 0.46
Previous RSV ^§^ infection prior to inclusion, *n* (%)	22 (28.9)	8 (25.0)	*p* = 0.68
**Baseline characteristics ^b^**			
First time wheeze, *n* (%)	15 (21.4)	6 (28.6)	*p* = 0.87
Hospitalized acute, *n* (%)	56 (80.0)	25 (83.3)	*p* = 0.70
RV at the emergency visit, *n* (%)	38 (48.1)	7 (21.9)	*p* = 0.011
RSV ^§^ at the emergency visit, *n* (%)	15 (19.2)	4 (12.5)	*p* = 0.40
Bacteria at the emergency visit, *n* (%)	35 (68.6)	16 (84.2)	*p* = 0.19
Eczema at inclusion, *n* (%)	18 (23.7)	6 (18.8)	*p* = 0.57
Positive Phadiatop (3-month follow up), *n* (%)	9 (13.2)	1 (3.3)	*p* = 0.17
Positive fx5 (3-month follow up), *n* (%)	14 (20.6)	8 (26.7)	*p* = 0.51
Vitamin D nmol/L, median (IQR)	83.5 (67.2–97)	81 (72–103)	*p* = 0.26
No RV at the emergency visit + negative Phadiatop at the 3-month follow up	32 (47.8)	21 (72.4)	*p* = 0.026
Eosinophils > 0.3 × 10^9^/L at the emergency visit, *n* (%)	9 (11.7)	3 (9.7)	*p* = 1.0
Neutrophils > 3.25 × 10^9^/L at the emergency visit, *n* (%)	66 (85.7)	26 (83.9)	*p* = 0.77
**Clinical data from the revisit at age 7 years ^c^**			
Self-reported food allergy *n* (%)	11 (13.9)	0 (0)	*p* = 0.032
Self-reported airborne allergy *n* (%)	20 (25.3)	0 (0)	*p* = 0.002
Asthma Control Test, median (IQR)	24.0 (22–26)	26.5 (26–27)	*p* < 0.001
BMI, median (IQR)	16.4 (15.3–18.1)	16.0 (15.4–17.4	*p* = 0.62
Eczema, *n* (%)	8 (10.5)	1 (3.4)	*p* = 0.44
FeNO, median (IQR) ^‡^	11 (7–15)	7 (6–9)	*p* = 0.05
FEV%, median (IQR)	87.3 (81.3–92.1)	91.2 (83.6–94.5)	*p* = 0.043
FEV1 reversibility test, *n* (%)	20 (27.0)	0	*p* = 0.003
Positive Phadiatop at 7 years, *n* (%)	23 (34.8)	3 (12.5)	*p* = 0.039
Positive fx5 at 7 years, *n* (%)	16 (24.2)	3 (12.5)	*p* = 0.23
Airborne allergy (symptoms + sensitization)	15 (22.7)	0 (0)	*p* = 0.009
Food allergy (symptoms + sensitization)	7 (10.8)	0 (0)	*p* = 0.18
Eosinophils > 0.3 at the 7 years follow-up, *n* (%)	32 (54.2)	7 (29.2)	*p* = 0.038
Neutrophils > 3.25 at the 7 years follow-up, *n* (%)	28 (47.5)	14 (58.3)	*p* = 0.37

^†^ Respiratory tract infection; ^‡^ *n* = 54; ^§^ respiratory syncytial virus; ^a^ cases without asthma at age 7 *n* = 31–33, cases with asthma at age 7 *n* = 72–80; ^b^ cases without asthma at age 7 *n* = 19–32, cases with asthma at age 7 *n* = 51–79; ^c^ cases without asthma at age 7 *n* = 11–33, cases with asthma at age *n* = 27–80.

**Table 3:** Cases without asthma vs. healthy controls at age 7 years.

Variable ^a^	Cases without Asthma at Age 7 *n* = 33	Healthy Controls *n* = 53	Statistics
Male, *n* (%)	23 (69.7)	38 (71.7)	*p* = 0.84
Heredity asthma and allergy *n* (%)	22 (68.8)	26 (50.0)	*p* = 0.092
Attend childcare or family home day care, *n* (%)	25 (78.1)	26 (49.1)	*p* = 0.008
>6 RTIs ^†^/year prior to inclusion, *n* (%)	22 (71.0)	6 (11.3)	*p* < 0.001
Previous RSV ^‡^ infection prior to inclusion, *n* (%)	8 (25.0)	2 (3.8)	*p* = 0.005
Reported food allergy at inclusion, *n* (%)	3(9.4)	1 (1.9)	*p* = 0.15
Eczema at inclusion, *n* (%)	6 (18.8)	2 (3.8)	*p* = 0.048
Positive Phadiatop (3-month follow-up), *n* (%)	1 (3.3)	1 (2.4)	*p* = 1.0
Positive fx5 (3-month follow-up), *n* (%)	8 (26.7)	8 (19.5)	*p* = 0.48
Vitamin D nmol/L, median (IQR) (3-month follow-up)	81.0 (72.0–103.0)	96.0 (76–107.7)	*p* = 0.27
Eosinophils > 0.3 × 10^9^/L at the emergency visit, *n* (%)	3 (9.7)	10 (19.6)	*p* = 0.35
Self-reported food allergy at age 7, *n* (%)	0 (0)	7 (13.2)	*p* = 0.04
Self-reported airborne allergy *n* (%)	0 (0)	0 (0)	
Eczema at age 7	1 (3.4)	1 (2.4)	*p* = 1.0
Positive Phadiatop at age 7	3 (12.5)	3 (12.0)	*p* = 1.0
Positive fx5 at age 7	3 (12.5)	10 (40.0)	*p* = 0.029
Airborne allergy (symptoms + sensitization)	0 (0)	0 (0)	
Food allergy (symptoms + sensitization)	0 (0)	2 (8.0)	*p* = 0.16
FeNO, median (IQR)	7.0 (6–9)	10.0 (6–15)	*p* = 0.21
Eosinophils > 0.3 × 10^9^/L, *n* (%)	7 (29.2)	6 (25.0)	*p* = 0.75
FEV%, median (IQR)	91.2 (83.6–94.5)	91.9 (86.4–98.8)	*p* = 0.68

^†^ Respiratory tract infection; ^‡^ respiratory syncytial virus; ^a^ healthy controls *n* = 15–53, cases without asthma at age 7 *n* = 11–33.

## Data Availability

Due to the nature of this research, participants of this study did not agree for their data to be shared publicly, so supporting data are not available.

## Supplementary Materials

The following are available online at https://www.mdpi.com/article/10.3390/children8060488/s1, Table S1: Inclusion and exclusion criteria for the GEWAC study, Table S2: Asthma definition at age 7 years and Table S3: Non-response analysis of cases who did not attend the revisit at age 7 years.
