# Supplementary material for: Early Life Wheeze and Risk Factors for Asthma—A Revisit at Age 7 in the GEWAC-Cohort
Source: Children (Basel). 2021 Jun 8;8(6):488. doi: 10.3390/children8060488 (PMC8229161; doi:10.3390/children8060488)
Supplement: Supplementary file 1 [file children-08-00488-s001.zip › children-1231865-SI.pdf]

Table S1. Inclusion and exclusion criteria for the GEWAC study.

	Inclusion criteria	Exclusion criteria
**Children with acute wheeze (cases)**	• Age 6-48 months<br>• Presenting at the emergency with acute symptoms of wheeze	• Prematurity (birth before 36 gestational weeks)<br>• Any chronic disease<br>• Any simultaneous complication such as bacterial pneumonia, sepsis, diabetes at the time of inclusion.
**Control group**	• Age 6-48 months	• Prematurity (birth before 36 gestational weeks)<br>• A history of bronchial obstruction/asthma<br>• Known sensitization to airborne allergens

Table S2. Asthma definition at age 7 years. Doctor's diagnosis of asthma as a compulsory criteria and at least one of the criteria: symptoms, medication or airway reversibility

**Criteria:**	
**DOCTOR'S DIAGNOSIS OF ASTHMA and at least one of following:**	
**1.Symptoms**	Self-reported asthma symptoms for 5 days or longer during the previous 12 months
and/or	
**2.Medication**	ICS[†] or leukotriene antagonist during 5 days or longer the previous 12 months
and/or	
**3.Airway reversibility**	Reversibility >12% in FEV1 after broncho-dilation

[†] Inhaled corticosteroids.

Table S3. Non-response analysis of cases who did not attend the revisit at age 7 years.

Variable[a]	Cases (non-response at the 7 year follow-up) *n* = 41	Cases (came to the 7 year follow-up) *n* = 113	Statistics
Male, *n* (%)	27 (65.9)	73 (64.6)	p=0.87
Age in months at inclusion, median (IQR)	19 (9.75-32.75)	19 (12-23)	p= 0.998
Exclusive breastfeeding 4 months, *n* (%)	17 (48.6)	66 (63.5)	p=0.12
Heredity asthma and allergy, *n* (%)	26 (72.2)	79 (73.8)	p=0.85
Maternal smoking during pregnancy, *n* (%)	5 (13.9)	9 (8.3)	p= 0.33
Eczema at inclusion, *n* (%)	4 (11.1)	24 (22.2)	p=0.15
>6 RTIs[†]/year prior to inclusion, *n* (%)	23 (63.9)	69 (65.7)	p=0.84
Rhinovirus at emergency visit, *n* (%)	20 (48.8)	45 (40.5)	p=0.36
Respiratory Syncytial virus at emergency visit, *n* (%)	7 (17.1)	19 (17.3)	p=0.98
Bacteria at the emergency visit, *n* (%)	13 (72.2)	51 (72.9)	p=0.96
Positive Phadiatop, *n* (%)	2 (7.4)	10 (10.2)	p=0.66
Positive fx5, *n* (%)	8 (29.6)	22 (22.4)	p=0.44
Eosinophils >0.3 at the emergency visit, *n* (%)	4 (10.3)	12 (11.1)	p=1.0
Neutrophils >3.25 at the emergency visit, *n* (%)	34 (87.2)	92 (85.2)	p=0.76
Days admitted to the hospital during the year after inclusion, mean (SD)	0.2 (0.7)	0.4 (0.9)	p=0.19
Number of admissions during the year after inclusion, mean (SD)	0.9 (1.6)	0.99 (1.7)	p=0.72

[†]Respiratory tract infection [a] Cases (non response at the 7-year follow-up) *n*=18-41 Cases (came to the 7-year follow-up) *n*=70-113.
